# The Inverse Association Between Cancer and Dementia: Is It Still Counterintuitive?

**DOI:** 10.1093/geroni/igaf122.538

**Published:** 2025-12-31

**Authors:** Michelle Shardell, Alan Rathbun, Shanshan Yao, Megan Marron, Venkatesh Murthy, Anne Newman, Eleanor Simonsick

**Affiliations:** University of Maryland School of Medicine, Baltimore, Maryland, United States; University of Maryland School of Medicine, Baltimore, Maryland, United States; University of Michigan, Ann Arbor, Michigan, United States; University of Pittsburgh, Pittsburgh, Pennsylvania, United States; University of Michigan, Ann Arbor, Michigan, United States; University of Pittsburgh, Pittsburgh, Pennsylvania, United States; National Institute on Aging, Baltimore, Maryland, United States

## Abstract

Epidemiologic studies have consistently demonstrated inverse associations between cancer and dementia. One hypothesis is that differential attrition bias leads to an apparent inverse association. Another hypothesis posits that cancer and dementia result from opposite extremes of shared biological mechanisms, where dysregulation in one direction promotes cancer risk, and dysregulation in the opposite direction promotes dementia risk. Such a shared mechanism would produce unmeasured confounding. To assess attrition bias, we used data from n = 2,153 participants in the Health, Aging and Body Composition Study who had neither a history of cancer (self-report) nor dementia (dementia medications or Modified Mini-Mental <78) at baseline to examine whether time-varying cancer history was associated with subsequent incident dementia using cause-specific discrete-time Cox models with and without inverse-probability weighting (IPW) for drop-out and death. To assess possible shared biological mechanisms, we used data from a subset of 1,674 participants with baseline plasma metabolomics characterized. We fit IPW cause-specific Cox models with and without adjustment for the top 20 metabolites jointly associated with cancer and dementia. Cancer history was inversely associated with dementia (Hazard Ratio[HR]=0.648; 95%Confidence Interval[CI]=0.441-0.951), which remained when using IPW (HR = 0.650; 95%CI=0.440-0.960). Among participants in the metabolomics subset, cancer history was inversely associated with dementia (HR = 0.620, 95%CI=0.391-0.982), but the association was slightly attenuated and lost statistical significance after adjustment for metabolites (HR = 0.669; 95%CI=0.420-1.065). Findings suggest that the inverse association between cancer and dementia is not explained by attrition bias, but may be partially explained by shared biological mechanisms (unmeasured confounding). Multi-omic replication studies are needed.

